# KISS1/KISS1R in Cancer: Friend or Foe?

**DOI:** 10.3389/fendo.2018.00437

**Published:** 2018-08-03

**Authors:** Stephania Guzman, Muriel Brackstone, Sally Radovick, Andy V. Babwah, Moshmi M. Bhattacharya

**Affiliations:** ^1^Department of Medicine, Robert Wood Johnson Medical School, Rutgers, The State University of New Jersey, New Brunswick, NJ, United States; ^2^Child Health Institute of New Jersey, New Brunswick, NJ, United States; ^3^Division of Surgical Oncology, The University of Western Ontario, London, ON, Canada; ^4^Department of Pediatrics, Robert Wood Johnson Medical School, Rutgers University, The State University of New Jersey, New Brunswick, NJ, United States; ^5^Cancer Institute of New Jersey, New Brunswick, NJ, United States

**Keywords:** kisspeptin, KISS1R, cancer, metastasis, prognostic marker, metastasis promoter, metastasis suppressor

## Abstract

The *KISS1* gene encodes KISS1, a protein that is rapidly processed in serum into smaller but biologically active peptides called kisspeptins (KPs). KISS1 and the KPs signal via the G-protein coupled receptor KISS1R. While KISS1 and KPs are recognized as potent positive regulators of the reproductive neuroendocrine axis in mammals, the first reported role for KISS1 was that of metastasis suppression in melanoma. Since then, it has become apparent that KISS1, KPs, and KISS1R regulate the development and progression of several cancers but interestingly, while these molecules act as suppressors of tumorigenesis and metastasis in many cancers, in breast and liver cancer they function as promoters. Thus, they join a small but growing number of molecules that exhibit dual roles in cancer highlighting the importance of studying cancer in context. Given their roles, KISS1, KPs and KISS1R represent important molecules in the development of novel therapies and/or as prognostic markers in treating cancer. However, getting to that point requires a detailed understanding of the relationship between these molecules and different cancers. The purpose of this review is therefore to highlight and discuss the clinical studies that have begun describing this relationship in varying cancer types including breast, liver, pancreatic, colorectal, bladder, and ovarian. An emerging theme from the reviewed studies is that the relationship between these molecules and a given cancer is complex and affected by many factors such as the micro-environment and steroid receptor status of the cancer cell. Our review and discussion of these important clinical studies should serve as a valuable resource in the successful development of future clinical studies.

## Introduction

Cancer is the second leading cause of mortatility and accounts for about 1 in every 6 deaths globally (http://www.who.int). At any time in development, the body contains abnormal cells with malignant potential or neoplastic characteristics. In the healthy host, these cells are detected, and destroyed by the immune system. However, under various physiological conditions these cells can escape the body's immune surveillance resulting in life-threatening tumorigenesis and deadly metastatic disease. The body produces molecules that can either inhibit

or promote the development of a tumor and its metastatic potential and identifying and studying these factors are essential in the treatment of cancer. In 1996, Welch and colleagues at The Pennsylvania State University College of Medicine, Hershey, Pennsylvania, conducted studies to identify the gene(s) responsible for the suppression of metastasis in chromosome 6/melanoma cell hybrids. Their brilliant work led to the discovery of a novel cDNA-designated KiSS-1 (1). This name was chosen to indicate that the cDNA encoded a metastasis suppressor sequence and also to recognize its discovery in the town of Hershey, Pennsylvania, the home of world-famous Hershey's chocolates known as Kisses. Out of that discovery and clever branding was born the era of kisspeptin biology.

In the original study from the Welch laboratory, Lee et al. (1) reported that among a panel of melanoma cells, KiSS-1 (later renamed *KISS1*) was expressed only in non-metastatic melanoma cells and when exogenous *KISS1* was expressed in C8161 melanoma cells, metastasis was suppressed in an expression-dependent manner. Thus, they concluded that *KISS1* suppresses the metastatic potential of malignant melanoma cell. Since this initial study (1), KISS1 (145 amino acid), the primary and full-length product of the *KISS1* gene and its derivatives (54, 14, 13, and 10 amino acids) called kisspeptins, as well as their cognate receptor KISS1R (previously called GPR54), have been reported to exhibit anti-metastatic and/or anti-tumoral roles in numerous cancers e.g., bladder (2, 3), ovary (4), colorectal (5–7), pancreas (8), pituitary (9), prostate (10), and thyroid (11), as previously reviewed (6, 12–14). Mechanistically, these molecules may exert their anti-metastatic effect on these cancers via a repression of matrix metalloproteinase MMP-9 activity and inhibition of cancer cell migration and invasion (14, 15). Interestingly, based on studies conducted on the human breast carcinoma MDA-MB-435 cell line, *KISS1* was also reported to suppress breast cancer metastasis (16). However, it is now recognized that MDA-MB-435 cells display a gene expression profile more closely resembling melanoma cancer cells, raising the possibility that these are melanoma and not breast cancer cells (17, 18). Based on the use of established cell models of breast cancer and breast cancer patient data, several studies from our laboratories (19, 20–22), and those of others (23–25) would later show that KISS1 and its receptor KISS1R, in striking contrast to most cancers, promote breast cancer invasion, and metastasis.

The finding that the same molecule can act as both a promoter and suppressor of tumorigenesis and metastasis is not unique to KISS1 or KISS1R as other molecules such as NFκB (26), c-Myc (27), AMP-activated protein kinase (AMPK) (28), transforming growth factor β (TGF-β) (29), SKY (30), and hyaluronidase (31) have been reported to play dual roles. Growing evidence suggest that the role any of these molecules assumes is dependent on many factors including the cancer type (e.g., melanoma vs. breast), stage of development (e.g., pre-malignant vs. malignant) and composition of its microenvironment (e.g., steroidal milieu and the absence and presence of other signaling molecules that might facilitate suppressor or promoter pathways). Thus, it is pivotal to study cancer in context (32). To date, in addition to playing a promoter role in breast cancer metastasis, KISS1 and KISS1R also appear to promote hepatic cell carcinoma (33).

Since the seminal discovery that *KISS1* regulates metastasis of melanoma cells (1), a large and growing body of studies has described roles for KISS1 and KISS1R mRNA and protein in regulating tumor growth and metastasis (6, 12–14). These studies are of tremendous importance as we forge forward toward developing these molecules as either prognostic markers and/or therapeutic targets in the treatment of various types of cancers. The goal of this review is therefore to summarize and discuss the key clinical evidence in support of the finding that KISS1 and KISS1R act as both promoter and suppressor of tumorigenesis and metastasis (Table 1).

**Table 1:** KISS1/KISS1R expression in patient tumors.

**Type of cancer**	**Clinical evidence: KISS1/KISS1R expression and plasma kisspeptin levels**	**Sample size**	**References**
Breast cancer	Increased KISS1 mRNA and protein in primary breast tumors and lymph node metastasis.	normal: 33 Tumor: 124	(34)
	Increased *KISS1* mRNA with the grade of the breast tumors; ERα-positive breast tumors; expressed sevenfold less *KISS1* than ERα-negative breast tumors.	Tumor: 92	(23)
	Increased KISS1/KISS1R protein vs. Control	Non-tumor: 11 (fibrocystic disease) Tumor: 43 resected breast adenocarcinomas	(35)
	KISS1 protein expression is positively associated with lymph node positive status.	Breast tumor microarray (*n* = 48)	(25)
Triple negative breast cancer (TNBC)	Increased KISS1/KISS1R mRNA and protein in primary TNBC tumors compared to healthy breast.	Healthy: 13 TNBC: 20	(19)
Hepatocellular Carcinoma (HCC)	Increased *KISS1R* mRNA expression in HCC vs. normal; no change in *KISS1* levels.	Normal: 8 HCC: 60	(33)
	KP-54 immunoreactivity associated with tumor grade, stage, poor prognosis	Normal: 10 HCC: 142	(36)
	Lower KISS-1 protein in HCC vs. normal.	Normal: 16 HCC: 150 patients	(37)
Pancreatic Cancer	High level of KP-54/KISS1R immunostaining associated with longer survival.	Tumors: 53 Normal: not reported	(38)
	Lower *KISS1* mRNA in pancreatic cancer tissue vs. control. Higher *KISS1R* expression in pancreatic tumors vs. matched controls.	Adjacent normal: 5 Tumors: 30	(8)
Colorectal Cancer (CRC)	Decreased *KISS1* expression associated with lymph node metastasis and poorer prognosis.	CRC:175	(5)
	Decreased expression of *KISS1/KISS1R* in tumor tissues compared with normal.	Normal: 80 CRC: 94	(6)
	Mean KP-54 (CRC): 86.2 ± 20.5 ng/ml; Mean KP-54 (control) patients: 49 ± 12.7 ng/ml	Controls: 59 CRC: 81	(39)
	Decreased *KISS1* mRNA expression and KP-54 protein expression - increased depth of invasion and lymph node metastasis.	Normal: 142 CRC: 126	(7)
Bladder Cancer	*KISS1* was found to be hypermethylated in ~83% of tumors, epigenetic-induced loss of *KISS1* expression associated with poor survival	804 primary bladder tumors	(2)
	Decreased or lost *KISS1* expression in invasive bladder tumors (*n* = 173) vs. controls	Normal urothelium: 25 Tumors: 173	(40)
Renal cell Carcinoma (RCC)	Higher *KISS1R* mRNA in RCC compared to non-neoplastic renal cortex	Non-neoplastic: 25 Tumors: 25	(41)
Ovarian Carcinoma	Lower KISS1 immunostaining in primary epithelial ovarian cancer (EOC) biopsies vs. control; patients with KISS1-negative tissues had a lower survival rate vs. to KISS1-positive patients	EOC: 207 Control: 60	(42)
	Favorable prognosis and overall survival associated with KISS1 and KISS1R immunoreactivity	Ovarian carcinomas tissue microarray: 518	(4)
	Median plasma kisspeptin: stage 1(*n* = 9): 17.4 pmol/L, stages 2-4 (*n* = 23): 7.8 pmol/L, control: 11.4 pmol/L	Cancer patients: 31 Healthy volunteers: 31	(43)

*Changes in the expression of KISS1/KISS1R transcripts, protein and circulating kisspeptins in cancer patients compared to healthy subjects*.

## Metastasis promoter roles

### Breast cancer

Breast cancer is the leading cause of cancer related deaths in women world-wide and metastasis is responsible for the majority of cancer deaths (44). Triple negative breast cancer (TNBC) comprises a heterogeneous group of tumors defined as a basal-like subtype lacking estrogen receptor alpha (ESR1), progesterone receptor (PGR), and human epidermal growth factor receptor 2 (ERBB2) (45). TNBC represents 15–20% of all breast cancers and occurs frequently in women under 50 years of age, in women with African ancestry or in Hispanic women. Unfortunately, these patients do not benefit from hormone receptor or HER2-targeted therapies, leaving chemotherapy as the main systemic treatment option (45). The prognosis for TNBC patients remains poor since patients are often high grade and either metastatic at time of diagnosis or succumb to metastasis within 3 years of diagnosis (45).

Spanning a little over one decade, several studies have highlighted a detrimental role for KISS1/KISS1R in breast cancer. Martin et al. (34) first demonstrated that KISS1 mRNA and protein levels were found to be elevated in ERα-negative invasive ductal carcinoma compared to ERα-positive tumors, demonstrating their positive correlation with tumor progression and poor patient prognosis. This study also revealed that breast tumors that were positive for lymph node metastasis showed higher *KISS1* levels compared to the lymph node negative tumors. Using immunohistochemistry, studies have shown that KISS1 and KISS1R is localized within the ductal carcinoma *in situ* and in invasive ductal carcinoma, and that there was higher cytoplasmic staining in tumors as well as surrounding cells, compared to normal tissue (19, 34, 35). Marot et al. (23) demonstrated that patients with high *KISS1* and *KISS1R* expression in breast tumors had the shortest relapse-free survival relative to tumors expressing low levels of these genes. This study showed that treatment of ERα-positive MCF7 and T47D breast cancer cells with tamoxifen, a selective estrogen receptor modulator (with antagonistic role in breast tissue), stimulated *KISS1/KISS1R* expression, implicating that ERα signaling downregulates KISS/KISS1R levels. This negative regulation of *KISS/KISS1R* expression by ERα is well-documented in the ARC KISS1 neurons in the hypothalamus (46). Along with the results reported by Martin et al. it appears that the increase in KISS1R correlates better with a metastatic capacity rather than with tumor growth. Next, Papaoiconomou et al. (35) found higher expression of KISS1 and KISS1R protein in breast cancer tissues (ductal carcinomas and lobar carcinomas) compared to non-cancerous fibrocystic mammary tissues. However, a significant correlation was not found between KISS1 and KISS1R expression and tumor grade, tumor size, lymph node positivity, histological type or ER status, results possibly due to the study's small sample size (*n* = 43).

Recently, we demonstrated that KISS1/KISS1R mRNA and protein expression was upregulated in primary TNBC tumor biopsies compared to healthy breast tissue (19) (Table 1). We found localization of KISS1 and KISS1R in invasive ductal carcinoma tumors using immunostaining. Furthermore, immunolocalization of endogenous KISS1R was found to be enhanced at the leading edge of migratory tumor cells (21). We also demonstrated that KISS1R signaling induces the drug resistant phenotype in TNBC cells, by inducing the expression of efflux drug transporter, breast cancer resistance protein (ABCG2) and by activating the tyrosine kinase, AXL (19). The P13K/AKT and Ras/ERK pathways are the most dysregulated signaling cascades in human carcinomas that are related to tumor drug resistance, survival, and proliferation (47). We found that KISS1R signaling in the drug-resistant cells led to an increase in the expression of AKT, ERK, and the anti-apoptotic protein, survivin. Specifically we and others found that ERα negatively regulates KISS1 (23), KISS1R expression and KISS1R-induced cell invasion (21). This may, in part, account for switching from the metastasis suppressor to metastasis promoter roles of the KISS1/KISS1R system in breast cancer. Thus, when ERα expression is lost (e.g., in TNBC), this results in increased transcription of KISS1/KISS1R, and increased receptor signaling and the induction of epithelial to mesenchymal transition (EMT), allowing epithelial cells to acquire invasive characteristics (14). KISS1R signaling stimulates TNBC cell invasion by inducing invadopodia formation by activating key invadopodia proteins, cortactin, cofilin, and membrane type I matrix metalloproteases (MT1-MMP), via a β-arrestin2, and ERK1/2-dependent mechanism (20). Additionally, KISS1R signaling can activate the epidermal growth factor receptor (EGFR) to promote TNBC invasion, via a β-arrestin2 and MMP-9 pathway (22). Interestingly, it appears that KISS1/KISS1R also mediate the effects of pro-invasive factors in TNBC, as it was recently reported that TGFβ-induced cancer cell invasion is dependent on KISS1 (25). Downregulation of KISS1 blocked TGFβ-mediated cancer cell invasion as well as MMP-9 expression and activity in TNBC cells, but not ERα-positive breast cancer cells. Through an immunohistochemical analysis of a tumor microarray, this study also showed that lymph node positive status is associated with high KISS1 protein levels.

In additional support for the pro-metastatic roles of KISS1R in breast cancer, a landmark study by Cho et al. (24) provided *in vivo* evidence that relative to wild-type mice, *Kiss1r* heterozygosity triggered a haploinsufficient phenotype where breast tumor initiation, growth and metastasis were delayed. This study also demonstrated that KISS1/KISS1R signaling occurs in an autocrine manner in breast epithelial cells to promote in breast tumor development in an animal model. Consistent with these findings, we observed that kisspeptin treatment of normal human mammary epithelial MCF10A cells induced cell transformation, resulting in a malignant phenotype. Kisspeptin treatment or exogenous expression of KISS1R in MCF10A cells induced EMT and stimulated cell invasiveness by inducing the expression of mesenchymal markers (N-cadherin, Snail/Slug) and loss of E-cadherin from cell-cell junctions (21). Finally, we also reported that in chick embryo assays, ERα-negative SKBR3 breast cancer cells expressing exogenous KISS1R exhibited increased invasion (21). Taken together, these *in vitro*, animal model studies and clinical findings provide substantial support that KISS1 and KISS1R promote metastasis in breast cancer.

### Liver cancer

Primary liver cancer is one of the leading causes of cancer deaths in the world where its incidence has increased by 75% between 1990 and 2015 (48). In the US, liver cancer cases have more than tripled since 1980, and cancer death rates have increased by almost 3% per year since 2000 (www.cancer.org), despite the reducing incidence of chronic hepatitis infections. Hepatocellular carcinoma (HCC) accounts for ~90% of primary liver cancers, and in the last decade the development of this cancer has been linked to obesity-related metabolic syndrome which is on the rise world-wide (49).

To date, few studies have examined the role of KISS1/KISS1R in HCC and the findings have been contradictory. Ikeguchi et al. (33) *first* showed that *KISS1* and *KISS1R* mRNA were overexpressed (22 and 43%, respectively) in surgically resected HCC samples, compared to non-cancerous liver and this was positively associated with disease progression and poor survival. Since none of the patients received preoperative chemotherapy or radiation therapy, the increase in *KISS1*/*KISS1R* levels was likely the direct result of the disease (33). In agreement with these findings, Schmid et al. (36) reported that in HCC patients who underwent liver transplantation had a worsened clinical outcome that correlated with elevated KP-54 expression, as assessed by immunohistochemistry. In contrast to these studies, Shengbing et al. (37) reported that KISS1 protein expression is lost in HCC, thus suggesting tumor suppressive roles in HCC. The authors examined the expression of MMP-9, a key driver of metastasis and report a negative association of KISS1 with MMP-9 in HCC (37). It remains unclear why the discrepancy exists among these studies, highlighting the need for further interrogation.

## Metastasis suppressor roles

### Pancreatic cancer

Pancreatic cancer remains a lethal disease since at the time of diagnosis most patients with pancreatic cancer have locally advanced tumors and/or metastases. While surgery represents the only curative treatment, just 10–15% of pancreatic cancer patients have resectable disease (50). Studies have shown that KISS1 and KISS1R are expressed in the pancreatic islets, in the endocrine alpha and beta cells, and regulates glucose stimulated insulin secretion (51, 52). Interestingly, in cancers such as pancreatic, ovarian, and colorectal cancer, a new facet in the relationship between KISS1/KISS1R and these cancers was uncovered. Namely, the expression of these proteins is high in the early stages of the disease but progressively become diminished with the advancement of the cancer. This observation is highlighted in some of the following studies but, as discussed, this requires further investigation.

Masui et al. (8) demonstrated that in pancreatic cancer patients, tumor *KISS1* levels were significantly higher compared to normal tissue (*n* = 30). The authors also compared *KISS1R* mRNA levels in cancer and matched normal tissues from each patient (*n* = 5) and found that the receptor expression was higher in cancer tissue compared to the adjacent normal in all paired samples. Nagai et al. made similar observations among 53 pancreatic ductal adenocarcinoma tissues, reporting strong immunostaining of KP-54 and KISS1R in tumors (38). They found that tumors that were negative for both KP-54 and KISS1R expression were significantly larger than tumors that were positive. Furthermore, they found that expression of KP-54 and KISS1R was associated with longer survival and recurrence was less frequent in patients who had KP-54-positive tumors compared with those who had KP-54-negative tumors. Plasma KP-54 levels were also measured in 23 patients; however, no significant difference in survival was found between the patients with high and low plasma levels. The lack of clinicopathological data for these patients as well as the absence of data on the KP-54 levels in healthy controls complicate the full interpretation of these findings.

Since KISS1 is expressed at reduced levels in advanced pancreatic cancer, McNally et al. (53) hypothesized that re-expression of *KISS1* would reduce metastases. Pancreatic cell lines such as BxPC-3, PANC-1 and SUIT-2 express low levels of endogenous *KISS1*, thus *KISS1* was overexpressed in a metastatic subclone of the SUIT-2 pancreatic adenocarcinoma cell line, S2VP10. SCID mice were implanted orthotopically with S2VP10L-KISS1 cells. Analyses of these mice revealed that mice bearing S2VP10L-KISS1 tumors developed fewer liver (98%) and lung (99%) metastases than control mice implanted with S2VP10L cells only expressing the empty KISS1 cloning vector. Based on the results, the authors concluded that KISS1 therapy might prove beneficial in suppressing the metastasis of pancreatic cancer.

It is important to note that the relationship between KISS1/KISS1R and pancreatic cancer requires further investigation as a study by Wang et al. (54) reported that the plasma KP-54 levels in pancreatic cancer patients were significantly higher when compared with healthy volunteers. However, a significant relationship was not found between KP-54 levels and clinicopathological factors such as tumor size, invasion, lymph node metastasis and distant metastasis.

### Colorectal cancer

Colorectal cancer (CRC) is the third most common cancer in Europe and the US, and 30% of patients have metastasis at diagnosis (55, 56). A decrease in this cancer mortality rate has been observed in countries where screening and improved treatments exist (57). Current treatments for metastatic colorectal cancer are considered palliative, but include combination of cytotoxic therapy as well as targeted therapy with anti-EGFR and anti-vascular endothelial growth factor. These treatments have significantly improved the progression-free survival of patients with metastatic CRC, which ranges from 22 to 29 months (58). However, many patients progress as tumors acquire resistance to therapies. Since CRC is a heterogeneous disease, there is a critical need for the discovery of novel biomarkers, to identify patients who will most likely benefit from treatment.

Okugawa et al. (5) conducted one of the first studies to determine the expression of *KISS1* mRNA and protein in 175 colorectal tumors and adjacent normal tissues from patients undergoing surgery, none of which received neo-adjuvant therapy. Kaplan-Meir survival studies indicated that patients with tumors with low *KISS1* mRNA expression had poorer prognosis and exhibited lymph node metastasis, in comparison to patients with tumors that had high *KISS1* levels. Using multivariate analysis, the authors concluded that decreased expression of *KISS1* is a significant independent prognostic marker. Although not quantified, immunodetection of KISS1 using an antibody raised against amino acids 46–146 revealed that KISS1 was highly expressed in primary CRC and in early stages of the disease and decreased in advanced stage-tumors. Canbay et al. (39) using a commercially available enzyme-immunoassay, measured plasma KP-54 levels in blood samples from 81 CRC patients and 59 age matched healthy controls. This study found plasma KP-54 levels were significantly higher in CRC patients (86.2 ± 20.5 ng/ml) than in controls (49 ± 12.7 ng/ml). They also found that KP-54 levels were significantly correlated with nodal involvement of CRC leading the authors to propose using plasma KP-54 as a predictive marker for lymph node metastases of CRC.

Ji et al. (6) examined *KISS1* and *KISS1R* transcripts in 94 samples from colorectal cancer tissues and 80 samples from normal tissue and found that the expression of *KISS1* had a negative correlation with Duke's staging, TMN staging, tumor size and lymph node metastasis. *KISS1R* was also reduced and low *KISS1R* expression was linked to poor prognosis in patients. Remarkably, patients who were undergoing chemo/radiotherapy showed a higher expression of *KISS1R* compared to the patients without radiotherapy. Mechanistically, KISS1 regulates CRC cell invasion by reducing the secretion and activity of MMP-9 in an ERK-dependent manner (6, 59). Chen et al. (7) also found that a decrease in *KISS1* expression and KP-54 protein expression correlated with an increased depth of invasion and lymph node metastasis in 126 CRC patients compared to 142 normal controls. To understand why *KISS1* expression was reduced, they investigated whether *KISS1* was inactivated by epigenetic mechanisms. By looking at the genomic methylation patterns of *KISS1*, they discovered hypermethylation of *KISS1* in 88.33% (105/126) of CRC samples; this was significantly higher than the rate observed in normal colorectal tissues (9/142). The DNA methyltransferase inhibitor azacitinde (5-Aza-2-deoxycytidine) was able to restore *KISS1* expression and the corresponding reduction of CRC cell invasion. These findings suggest that *KISS1* is epigenetically modified in CRC, however further studies are required to better understand the potential role of *KISS1* hypermethylation in the progression of colorectal cancer.

### Bladder cancer

Urinary bladder cancer is the ninth most common malignant disease and the 13 most common cause of cancer-related death in the world. In bladder cancer, *in situ* hybridization studies revealed that *KISS1* expression was significantly decreased or lost in invasive bladder tumors (*n* = 173) compared with their respective normal urothelium (*n* = 25) (40). Patients (*n* = 69) with lower *KISS1* expression in bladder tumors showed a significant association with worse overall survival. *KISS1* expression was significantly lower in bladder tumors with vascular invasion compared with normal urothelium. Furthermore, in this study, the authors observed that all bladder tumors developing distant metastases showed a complete loss of *KISS1*.

As observed in CRC, *KISS1* was found to be hypermethylated in over 83% of 804 primary bladder tumors (2). This study also revealed that the epigenetic-induced loss of *KISS1* expression was associated with poor survival and suggested that *KISS1* levels have predictive value in identifying patients with poor outcome. Takeda et al. (3) examined KISS1 and KISS1R expression in 151 bladder cancer patients to determine their prognostic significance and reported that KISS1 immunoreactivity was significantly decreased in advanced stages of bladder cancer and inversely associated with tumor grade and stage. However, no association in KISS1R expression was found with disease progression. Moreover, this study demonstrated that KP-54 treatment significantly reduced the invasiveness of bladder cancer cells and lung metastasis in a metastasis animal model by inhibiting the expression and activity of MMP-9 via blockage of the nuclear translocation of NF-kB, a transcriptional regulator of MMP-9. Thus, this study provided pre-clinical evidence that KP-54 treatment may be an effective inhibitor of metastasis in urothelial carcinoma. Multivariate analysis revealed that KISS1 expression was an independent predictor of bladder cancer metastasis and overall patient survival. Thus, these studies strongly suggest that KISS1 expression in bladder tumors might be a biomarker of disease, especially for predicting the occurrence of metastases in highly aggressive urothelial carcinoma.

### Ovarian cancer

Ovarian cancer is a commonly diagnosed cancer worldwide and causes more deaths than any other cancer of the female reproductive system. It ranks among the top five deadliest cancers in most countries (48) and in the United States alone, each year about 20,000 women are diagnosed with ovarian cancer (60). KISS1 expression levels have also been linked to survival in ovarian cancer patients as reported in several studies. Hata et al. (61) observed that ovarian cancer patients exhibiting elevated levels of *KISS1/KISS1R* expression, as detected by quantitative PCR, had favorable prognosis using COX regression analysis in a cohort of 76 patients. Cao et al. (62) reported that *KISS1* expression was significantly higher in 40 pre-operative epithelial ovarian cancer (EOC) primary tumors compared to 20 uterine fibroids used as normal tissue. The presence of metastasis and tumor size was negatively associated with pre-operative *KISS1* expression; patients with low *KISS1* expression had shorter survival time than those with high expression. (62).

In addition to the mRNA expression studies described above, protein levels were also assessed in primary ovarian tumors. Interestingly, while the former study reported high *KISS1* levels in pre-operative EOC primary tumors, Yu et al. (42) found that KISS1 immunostaining was much lower in 207 primary EOC biopsies compared to control biopsies (60 benign tumors, serous- or mucinous-cystadenoma). Nevertheless, Kaplan-Meir survival data indicated that patients with EOC KISS1-negative tissues had a lower survival rate compared to KISS1-positive patients, and among these patients, KISS1 protein expression was inversely associated with tumor grade and stage. Similarly, in the largest study of ovarian biopsies done to date, Prentice et al. (4) conducted an immunohistochemical analysis of KISS1 and KISS1R on a tissue microarray consisting of 518 ovarian carcinomas and found that strong KISS1 and KISS1R immunoreactivity was significantly associated with favorable prognosis and overall survival.

To determine whether circulating levels of plasma kisspeptins are dependent on the stage of ovarian cancer, Jayasena et al. (43) measured the levels of plasma kisspeptin concentration in 31 patients with ovarian carcinoma (Stages I to IV) and 31 healthy volunteers, using an in-house radioimmunoassay. They found that the mean kisspeptin concentration in the patients with stage I was significantly higher, compared with the patients with ovarian carcinoma of stages 2 to 4 vs. controls: 25.1 ±15.2) pmol/L (stage 1), 11.8 ±10.3 pmol/L (stages 2 to 4), and 13.1 ±6.92 pmol/L (controls). Thus, stage 1 patients had an increased plasma level compared to the control and stages 2–4. Larger studies are required to further examine the relationship between plasma kisspeptins and ovarian cancer. Based on the various ovarian cancer studies, the independent findings propose that KISS1/KISS1R protein levels could be used as prognostic biomarkers of disease progression in ovarian cancer.

### Prostate cancer

Prostate cancer is the second leading cause of cancer mortality in men of 40 years of age and older (63). Currently, chronically administered gonadotropin-releasing hormone receptor (GnRH-R) agonists, which induce androgen deprivation, represent the primary clinical tools used for treating prostate cancer (64). Kisspeptin is powerful trigger of GnRH secretion and thereby a key positive regulator of the hypothalamus-pituitary-gonadal (HPG) axis. Studies have shown that administration of potent and long-acting kisspeptin agonists decreased serum testosterone levels by suppressing the HPG axis in rats and humans (65–67). Thus, it would be interesting to see if treating patients with kisspeptin agonists and antagonsits results in regression of prostate tumors and better outcome.

To determine whether KISS1 also has direct anti-metastatic activity in prostate cancer cells, Wang et al. examined KISS1 protein expression in 253 prostate tissue samples (normal tissue and prostate cancer) and found that KISS1 expression correlated negatively with clinical staging (10). Interestingly, in another study it was observed that there was no significant difference in plasma kisspeptin levels in 92 prostate cancer patients compared to healthy subjects (68). In addition to the prostate tissue samples, Wang et al. also measured *KISS1* and *KISS1R* expression in metastatic human prostate cancer cell lines and observed that decreased mRNA expression correlated with increased metastatic ability of these cancer cell lines (10).

To understand how kisspeptin might exert direct anti-metastatic effects in prostate cancer, KISS1 was re-expressed in PC3M cells that lack KISS1 and this resulted in an inhibition of cell migration and invasion and re-sensitization of cells to chemotherapeutics (10). Another study showed that KISS1R signaling induced the activation of eukaryotic translation initiation factor 2α kinase 2 (EIF2AK2) in prostate cancers and thereby inhibited cell growth and metastasis (69). Clearly, kisspeptin can act on prostate cancer cells both indirectly via the HPG axis and directly, thus its clinical value in treating prostate cancer is very promising and awaits further studies.

### Lung cancer

Lung cancer is the most commonly diagnosed cancer and the leading cause of death in men (57). Zheng et al. showed that the expression of *KISS1* was higher in stage I-II compared to stage III-IV and therefore showed an inverse relationship between *KISS1* expression and progression of non-small cell lung cancer (NSLCL). *KISS1* expression was also higher in the primary tumors compared to the second metastatic site, again showing that *KISS1* functions as a metastasis suppressor (70). In another study by Sun et al. the authors analyzed the expression of KISS1 and KISS1R in 28 patients and found that KISS1/KISS1R expression was higher in stage III compared to stage IV, again demonstrating an inverse relationship between KISS1/KISS1R expression and progression of NSCLC (71). Karapanagiotou et al. measured circulating levels of kisspeptin in 96 NSCLC patients (76 with metastatic disease and 21 with locally advanced disease) and detected no difference in plasma kisspeptin levels between NSCLC patients and healthy volunteers or between locally advanced and metastatic disease patients (72). The finding is reminiscent of that seen in prostate cancer where at the cellular level while KISS1 and KISS1R expression correlated with the disease, serum kisspeptin did not (10, 68).

## Concluding remarks and perspective

KISS1 and KISS1R clearly play important roles in regulating the progression of cancers. However, the roles (that is, suppressor or promoter) these molecules play are cancer context-specific and may be further modulated by other factors such as the micro-environment. Thus, as highlighted in this review, the discrepancies reported among some studies analyzing the same cancer type is not entirely surprising. Discrepancies may also be the result of different experimental procedures and use of different reagents. In particular, the use of different antibodies might be a major culprit. It is well known that many of the commercially-available KISS1R antibodies in use are not highly specific and thus, if proper and detailed antibody control studies are not conducted, some results might be incorrectly interpreted. For example, antibody specificity should be verified using knock-out and/or knock-down approaches, as we have employed in our studies (20). Additionally, it is important to know whether a given antibody detects the full length KISS1 or smaller peptides. It remains possible that the full-length protein might have one relationship with the given cancer (for e.g., high KISS1 is associated with higher survival rates) while the peptides may have the opposite relationship. Thus, in addition to assessing protein levels, it is important to investigate changes in *KISS1*/*KISS1R* transcript levels as well. Positive correlation between mRNA and protein data strengthens a given study. The lack of clinicopathological parameters such as whether patients received neo-adjuvant chemotherapy or whether the primary tumor was surgically removed or left in place can also greatly complicate the interpretation of the results leading to discrepant findings and conclusions. Additionally, the size of clinical cohorts remains a great challenge and care must be placed on the over-interpretation of data based on small clinical populations. Nevertheless, even small cohorts have their utility in helping to design larger cohort studies.

To summarize, in the cancers where KISS1 is thought to function as a tumor suppressor, clinical analysis of KISS1/KISS1R expression patterns suggests two general scenarios. It is possible that there is an initial upregulation of KISS1/KISS1R expression to suppress tumor progression (for example, as seen in pancreatic and ovarian cancers). KISS1/KISS1R expression is high in early stages of the disease and patients with high gene expression had longer survival and better prognosis. In colorectal, bladder and renal cell carcinoma, KISS1 expression is decreased (likely due to hypermethylation), and this was associated with lower survival rates and increase in metastasis. In breast cancer, such as TNBC where ERα is lacking, KISS1/KISS1R expression is elevated in tumors, and this pathway appears to promote tumor growth and metastasis and is associated with poor patient outcome. However, several questions remain unanswered. For example, does circulating plasma kisspeptin modify cancerous tissue? Do tumors produce kisspeptin *in situ*? Is the expression of KISS1/KISS1R associated with an earlier or later development of cancer? Do normal cells produce kisspeptin to minimize metastasis? Further studies are warranted to study the KISS1/KISS1R signaling pathway in each cancer type.

In conclusion, despite the limitations of some clinical studies, it remains abundantly clear that KISS1 and KISS1R play important roles in regulating the progression of cancers and the molecules have the strong potential to be used in developing novel therapies and/or as prognostic markers in treating cancer, the second most deadly disease worldwide.

## Author contributions

MoB is the senior author on this manuscript. She helped with manuscript preparation and identification of the topics to cover. SG wrote the manuscript. SR, MuB, and AB assisted with manuscript preparation and provided critical feedback, especially with the clinical aspects.

### Conflict of interest statement

The authors declare that the research was conducted in the absence of any commercial or financial relationships that could be construed as a potential conflict of interest.

## Abbreviations

AMPK: AMP-activated protein kinase
BCRP: Breast cancer resistance protein
CRC: Colorectal cancer
DAG: Diacylglycerol
EGFR: Epidermal growth factor receptor
EMT: Epithelial-to-mesenchymal transition
ESR1: Estrogen receptor alpha
EOC: Epithelial ovarian cancer
HER2: Human epidermal growth factor receptor 2
HCC: Hepatocellular carcinoma
HPG: Hypothalamus-pituitary-gonadal
KP: Kisspeptin
KISS1R: Kisspeptin receptor
MMP: Matrix metalloproteinases
PGR: Progesterone receptor
RCC: Renal cell carcinoma
TGF-β: transforming growth factor β
TNBC: Triple negative breast cancer.
